# Patients’ Experience of Attending a Binge Eating Group Program – Qualitative Evaluation of a Pilot Study

**DOI:** 10.1177/23779608211026504

**Published:** 2021-06-30

**Authors:** Kristine Rørtveit, Bodil Furnes, PhD, Elin Dysvik, PhD, Venke Ueland, PhD

**Affiliations:** 1 Department of Research, Stavanger University Hospital, Stavanger, Norway; 2 Faculty of Health Sciences, University of Stavanger, Stavanger, Norway

**Keywords:** affect consciousness, binge eating disorder, cognitive therapy, focus group, therapeutic writing, qualitative design

## Abstract

We developed a group program for patients with binge eating disorders (BED), comprising cognitive therapy, affect consciousness, and therapeutic writing. We wished to investigate how therapeutic writing and affect consciousness were experienced by the patients when integrated in a cognitive behavioral therapy (CBT) program. To our knowledge, such an intervention has not been tried in patients with BED. Aim: To explore patients’ experience of attending a binge eating group program comprising therapeutic writing, affect consciousness, and CBT. Research question: How do patients evaluate their experience of attending an integrative binge eating group program? Method: A qualitative design using an evaluative focus group interview with participants (four women and two men) who had completed the pilot program. Results: Three themes emerged: *Enhanced self-awareness about the meaning of feelings*; *A more generous attitude towards oneself*; and *On the path to a better grip on the eating difficulties*. Discussion: We interpreted the three themes in light of transition processes. The program was described as an essential part of the healing process and seems valuable for enabling new approaches leading to therapeutic changes when suffering from BED.

## Introduction

According to the Diagnostic and Statistical Manual of Mental Disorders (DSM-5) (American Psychiatric Association, 2013) binge eating disorder (BED) is associated with recurrent episodes of binge eating, which lead to distress and feelings of disgust, depression, and/or guilt. The eating episodes typically occur in a discrete period of time (e.g., within any 2-hour period), during which abnormally large amounts of food are consumed despite not feeling physically hungry, eating until uncomfortably full while not being physically hungry, and/or eating alone because of shame and embarrassment. The amount of food consumed is larger than what others would eat and a sense of lack of control over eating is typical. When the episodes occur at least one day per week for three months, the DSM-5 criteria for a BED diagnosis are fulfilled. No compensatory behaviors are associated with BED such as purging, fasting, or excessive exercise (American Psychiatric Association, 2013).

The reasons for BED are complex (Fairburn et al., 2003) and its development is characterised by background conditions, triggering circumstances, and maintaining factors (Buhl, 2002; Reijonen et al., 2003). BED and binge eating episodes are associated with powerful negative feelings (Citrome, 2017; Dingemans et al., 2009; Duarte et al., 2016; Fox et al., 2016; Nicholls et al., 2016) such as disgust, depression, and guilt (American Psychiatric Association, 2013). Difficulties in emotional regulation (Kukk & Akkermann, 2017) and an association with severe mental health problems such as depression and risk of suicidal ideation (Baek et al., 2018; Brown et al., 2018; Smith et al., 2017; Ural et al., 2017) are common. The existing literature calls for more mental health care and research on binge eating therapies (Brown et al., 2018; Grenon et al., 2018).

In the absence of a medical cure for many patients suffering from binge eating problems, it is crucial to find non-pharmacological strategies and powerful ways to handle BED. Cognitive behavioral therapy (CBT) in groups is the preferred approach for reducing eating disorder symptoms (Fairburn, 1995; Fairburn & Harrison, 2003; National Institute for Health and Care Excellence (NICE), 2017), consisting of psychoeducation; self-monitoring; analysing problems and goals; food intake planning; and body exposure training. Recently, mindfulness-based interventions for disordered eating have increased and it is concluded that mindfulness-based interventions can be considered effective for reducing binge eating (Godfrey et al., 2015). However, new interventions need to be developed in order to address the variations in the underlying psychology of BED and the consequences for mental health (Brown et al., 2018). Affect integration, defined as “the functional integration of affect in cognition, motivation and behaviour” (p. 27) is emphasized across different therapeutic models (Solbakken et al., 2011) and is a tool that focuses on the mental struggle and different sets of feelings. Several studies also point to writing as a therapeutic tool for managing and adjusting to demanding and challenging life experiences such as traumatic, stressful or emotional events (Baikie & Wilhelm, 2005; Smyth et al., 2001). Such writing has the potential for positive health gain (i.e. managing chronic pain), increased well-being, and even for strengthening the immune system (Furnes & Dysvik, 2011, 2012; Lowe, 2006; Pennebaker, 2000). Moving from the experience of suffering to the experience of health is a transition process, which may be challenging because it can lead to vulnerability and affect health (Meleis, 2000, 2010).

In the present pilot study, we explore different aspects of a binge eating group program (BEGP) implemented as group therapy in a natural clinical setting, with focus on deeper reflections about BED. A BEGP that comprised CBT (Fairburn, 2008), affect consciousness (Solbakken et al., 2011), and therapeutic writing (Furnes & Dysvik, 2012) was developed.

### Implementing the Binge Eating Group Program

#### Description of the Intervention

The BEGP (Table 1) took place for 10 weeks, with weekly 3-hour meetings and included CBT and psychoeducation. The CBT-model was based on a Norwegian translation of the handbook on the CBT eating disorders model (Fairburn, 1995; Fairburn & Sjøbu, 2014), which targeted eating patterns, thoughts, feelings, and typical binge eating risk-situations. The focus was motivation for changing eating behavior (Fairburn, 2008). The participants were asked to read the handbook prior to the program. During the program they were introduced to psycho-education and homework-exercises, which are typical CBT-activities. Homework involved making notes and keeping a diary of eating patterns.

**Table 1.** The Binge Eating Program.

Structure and content of the 10 session program in groups: Weekly meetings, each session 3 hours, (1) Introduction and reflection about the previous session – lunch break, (2) Mindfulness and therapeutic writing exercise – coffee break, (3) Reflection and evaluation			
Session	Psychoeducation (cognitive focus)	Therapeutic writing exercise (affect focus)	Homework, document the following when writing your meal time diary:
1	Regular eating and cognitive model	Interest, joy, and comfort.*Write about: Typical situations; bodily sensations; their meaning; your reactions; your non-verbal and verbal expression of feelings.	How you plan your meals Monitor eating and meals: what you eat, time, place and if you binge eat
2	Risk situations, meal-advice, the paradox of dieting	Anxiety, fear*	Your typical risk situations What risk situations are associated with eating and meals, what food leads to a risk of binge eating, do time and location play a role and what are your thoughts in such risk situations?
3	Anxiety, stress, coping strategies	Irritation/anger/rage*	How you cope with stress Is stress associated with eating and meals: what foods do you eat when stressed, do time and place play a role, does stress lead to binge eating episodes, and what are your thoughts when stressed? What coping strategies do you employ?
4.	Dysfunctional thoughts	Guilt*	Your dysfunctional thoughts Do you have dysfunctional thoughts about eating and meals that trigger binge eating episodes?
5	Triggers of binge eating	Shame*	How do you deal with triggers? Are the triggers associated with what you eat, the time and place of meals, binge eating episodes, and what are your thoughts about them?
6	Ways of handling impulsesProblem solving strategies	Disgust/abhorrence*	Your problem solving pattern What strategies do you employ to manage/cope with impulses related to eating and meals?
7	Ways of handling feelings	Contempt*	How you feel when you binge eat? Have you any strategies for managing your thoughts and feelings in such situations?
8	Handling feelings and conflicts	Sadness/despair*	Your way of handling feelings and conflictsHow do you manage feelings and conflicts related to eating and meals? What are your thoughts, conflicts and feelings about binge eating episodes?
9	Confirming thoughts and feelings	Envy/jealousy*	Thoughts and feelings in a binge eating situation How do you manage feelings related to eating and meals? What are your feelings about binge eating episodes?
10.	Affect integration and binge eating	Care/closeness/devotion*	Are you aware of how you manage relapses and strong feelings?

*Write about: Typical situations; bodily sensations; their meaning; your reactions; your non-verbal and verbal expressions of feelings.

In this program, the participants were also introduced to therapeutic writing, which aimed to provide a tool for self-investigation (Furnes & Dysvik, 2012). These exercises focused on different sets of feelings, and the integration of awareness, senses, toleration, and the expression of them (Solbakken et al., 2012). The affect regulation and therapeutic writing targeted in-depth processes for understanding underlying feelings associated with and that had an impact on binge eating.

The detailed program was pre-designed by the first author (K.R.) in collaboration with the research group. One specific writing exercise was given in each session.

(Please see Table 1: The binge eating group program).

We were curious about how therapeutic writing and affect consciousness were experienced by the patients when integrated in a CBT-model (Fairburn, 2008). To our knowledge, such an intervention has not been tried in patients with binge eating, nor has previous research documented experiences of affect consciousness implemented with CBT. The present pilot study is a part of a larger study, which investigates different elements of the program. The pilot study provides a qualitative evaluation of the participants’ experiences of attending such a program.

### Aim and Research Question

The aim was to explore (Rørtveit et al., 2020) patients’ experience of attending a binge eating group program comprising therapeutic writing, affect consciousness, and cognitive therapy. The research question was: How do patients evaluate their experience of attending an integrative binge eating group program?

## Methods

We used a phenomenological-hermeneutic approach inspired by Ricœur (Delmar et al., 2006; Dossey & Keegan, 2016; Furnes, 2008; Furnes & Dysvik, 2011; Ricœur, 1976), which enables exploration of the experiences of participating in a BEGP in groups. Data was collected by the means of a focus group interview in order to stimulate the discussion and open up new perspectives (Bradbury-Jones et al., 2009). This approach can increase understanding of the investigated area at a deeper level, as individual experiences are captured by the in-depth dialogue.

### Participants and Setting

A purposive sample (Polit et al., 2001) was chosen, as the most appropriate way to obtain knowledge about the investigated areas was to select patients from the current BEGP. We requested permission to include patients who had completed the pilot group intervention in the group-therapy ward during the period 2017–2018. The pilot study was carried out in a hospital on the West Coast of Norway in collaboration with the University of Stavanger. The necessary prerequisites, such as a room for group therapy, three professional therapists and time to perform the intervention were obtained. Potential participants were identified by the therapist and invited to participate by the secretary at the ward, who also informed them about the pilot study. All who were invited agreed to participate. The focus group, which was audio-taped and transcribed verbatim by the first author (K.R), took place in December, 2018.

Inclusion criteria: The included participants had fulfilled the BED diagnosis criteria and attended the BEGP > 80% presence, >18 years old, able to speak and understand the Norwegian language, non-psychotic and had no drug addiction.

Exclusion criteria: Participants were excluded if they had severe mental disorders, ADHD or other symptoms that could have a negative impact on the group dynamic.

(Please see Table 2: Characteristics of the sample)

**Table 2.** Characteristics of the Sample.

	Age	Gender	Work/school status	Relationship status	Previous weight-related treatment
					
P1	50	F	Employed	Single 2 adult children	Gastric Sleeve 2016, BMI: 42.1
P2	61	F	Employed	Single Married and divorced twice 2 adult children	Applied for bariatric surgery. Withdrew before surgery after attending the group. BMI 42
P3	23	M	Unemployed/ Student	Single	No previous weight-related treatment. BMI: 46.1
P4	42	F	Employed	Single, divorced 1 adult daughter	Applied for bariatric surgery. BMI 37
P5	39	M	Employed	Single	Applied for bariatric surgery. BMI: 47.3
P6	50	F	Employed	Divorced remarried. 2 children	Gastric Sleeve surgery spring 2018 (just before the group started) BMI: 42.2

Two men and five women aged between 23 and 61 years agreed to participate (Table 2). They had already been approved to attend the program and all members of the same group agreed to participate in the focus group. We did not investigate whether the differences in the participants’ responses were related to their background or age as their individual needs varied due to the degree of symptom intensity and loss functional level. They had different histories of lifestyle-changing treatment, diets, and bariatric surgery. Their binge eating ranged from daily bingeing and excessive eating to continuous snacking; all had extensive mental health issues such as serious body dissatisfaction, depression, shame, and periods when they lost control over their food intake. Their Body Mass Index ranged from 35–40 (normal: 19–25) and when looking back on their BED history they reported a duration of 10–14 years.

### The Researchers’ Pre-Understanding and Relationship to the Participants

The first author (K.R.) has a scientific background and long clinical experience as a psychiatric group therapist, with expertise in group therapy targeting different mental health areas (Rørtveit et al., 2010; Rørtveit & Severinsson, 2012). K.R. ran the therapeutic groups as well as the evaluation focus group. The second and third authors (E.D., B.F.) have long academic experience of developing nursing interventions related to CBT and therapeutic writing (Dysvik & Furnes, 2018; Gjesdal et al., 2019). The last author (V.U.) has long academic experience of caring and hermeneutic text reading (Ueland et al., 2019). The three latter authors did not know the participants and the last author performed the initial analysis of the transcribed text.

### Data Collection

One focus group interview (Colucci, 2007; Morgan, 2010) was performed with six patients a short time after the pilot intervention was completed. The interview focused on qualitative, open-ended evaluative questions (Morse et al., 2000). A focus group was considered valuable as the patients were familiar with each other due to having participated in the same group program. Focus groups are often used when group dynamics on one specific topic are desired (Tenny et al., 2020). We wanted to generate information on a collective view as well as a rich understanding of the participants’ experiences of and beliefs (Gill et al., 2008) about the program. In our case, the researcher (first author) was the moderator as she conducted the focus group and explained the research topic to the group. The co-moderator was a group therapist from the ward. As the evaluation provided rich and important data, we deemed it appropriate to publish it as a pilot study. The following open-ended questions were formulated and follow-up questions such as ‘Can you please tell me more about that?’, ‘Do you have any examples?’, and ‘Can you please describe the latter again?’ were used to clarify and deepen the responses:

- What were your experiences of the current group program; how did it affect you and your eating problems?
- Please describe any specific experiences of the cognitive therapy, affect focus and/or therapeutic writing when attending the program.

### Data Analysis and Interpretation

The phenomenological-hermeneutic approach guided the interpretation of the data in accordance with our pre-understanding of the phenomenon and context (Delmar et al., 2006; Furnes, 2008; Furnes & Dysvik, 2011; Ricœur, 1976). According to Ricœur, reading the text is a dialectic movement between the attitudes of ‘explanation’ and ‘understanding’, where understanding and explanation may overlap and interact with each other (Ricœur, 1976).

*Naïve reading* was undertaken to gain an overview of the text and a holistic understanding of the meaning content of ‘what is said’ (manifest meaning).The qualitative text was read, re-read, and worked with thematically in relation to the research question. In such an approach the language and spoken word constitute the medium that forms the basis for the interpretation. Quotations from the participants were analyzed. The last author (V.U.) performed the first step of the analysis of the text, as she was not familiar with the participants. She analyzed the text in a stepwise manner, after which the whole team reached agreement on the themes.

*Structural analysis* was undertaken in accordance with Ricœur (1976), as the intention of a structural analysis is to make a deeper critical interpretation possible by clarifying the dialectic between the holistic understanding (‘what is said’) and an explanation of the text (‘what is spoken about’) (Delmar et al., 2006). Interpretation of the explanatory structures and an understanding of the content will lead to the development of themes.

*Critical interpretation and discussion* took place as the themes were interpreted according to the whole, with reference to relevant theory or previous research. The naïve reading and structural analysis guided the selection of theory to illuminate and theorize the findings.

Please see Table 3: Examples of the steps of the analysis covering three themes.

**Table 3.** Example From the Analysis Steps Covering Three Themes.

Enhanced self-awareness about the meaning of feelings		A more generous attitude towards oneself		On the path to a better grip on the eating difficulties	
Units of meaning (What is said?)	Units of significance (What is being spoken about?)	Units of meaning (What is said?)	Units of significance (What is being spoken about?)	Units of meaning (What is said?)	Units of significance (What is being spoken about?)
Shaking things up helps. I had an aha-experience (P5). Instead of confronting him (husband) when I got mad at him, I would eat a bag of chips. Not only when I was at home, but wherever I was. However, telling others about it (the feeling), putting a name on it, has a special power in itself (P6).	Having to focus on feelings was demanding, but at the same time, it was something that strengthened the individual’s idea of her/himself. Recognizing one’s own emotions and telling others about them was seen as something that strengthened the individual’s idea of her/himself.	Seeing things in a longer perspective. Where have I come from and where am I now (P3)? It was just a small setback. You’ve not fallen off. It’s not a disaster if you fail on one single day. Ok. It was one of those days. Tomorrow I’ll be back on track (P6).	They did not have to give up if they failed to succeed in meeting their expectations every single day. Instead, they had learned to find possible alternative thought patterns as setbacks were accepted as part of their suffering.	There has been a change. I have a plan when I go to the store (P3). I don’t find it easy, but I feel quite happy inside and I’ve learnt a few things about how to handle it (P4). I try to live in the present rather than floating along with one foot in the past and one in the future, not present in the here and now (P3).	It was as if they had gained a more positive grip on their ED, which helped them to lead a more satisfying life. It felt good to glimpse a way of coping, setting the course ahead, instead of resisting the process.

### Ethical Considerations

This pilot study adheres to the general ethical principles contained in the declaration of Helsinki (World Medical Association, 2008). Ethical approval was granted by The Regional Ethics Committee (No. 2018/1773-1) and the Head of the Research and Human Resources Department at the hospital.

## Results

The variation of the participants’ experience of problems related to weight and eating was rich. The duration of their bodily problems related to food and dieting indicated that they all had a long history of trying to do something about their weight. They were eager to reflect at a deep level in order to understand their own eating condition. The BED ideation was new to all of them, which was an interesting aspect because they all mentioned experiences of becoming more aware during the therapy sessions. All participants expressed relief that by attending the evaluation their experiences could be useful to somebody else. The analysis of the transcribed material revealed three perspectives on how the participants evaluated the BEGP. The three themes were:

1. *Enhanced self-awareness about the meaning of feelings*
2. *A more generous attitude towards oneself*
3. *On the path to a better grip on the eating difficulties*

### Enhanced Self-Awareness About the Meaning of Feelings

The participants all expressed and reflected on how participating in the BEGP led to new awareness about feelings. They became more familiar with their own feelings and saw themselves more clearly.

*I’ve been through some periods of binge eating. It was a very negative experience. I felt sad and I knew what I was. Sad. I gave it a name (P4).*

They described how the BEGP triggered a consciousness-raising process. Writing also helped them to name their different feelings and get to know themselves. Some found it challenging to focus on their current feeling, but at the same time they reflected on the benefit and necessity of becoming more aware of their own feelings:

*What I really dreaded was: what is the specific feeling today. Then I gave in to it a bit (P2).*

*Normally I’d rather not think about feelings. The program almost forced us to confront them (P6).*

*I was to write about feelings. That calmed me somehow. So I started. That felt good (P3).*

*We are to address issues, not merely collect things (P3).*

*But then I was confronted with feelings that were hard to grasp. Are you on the right track? Is it that kind of feeling or something else (P5)?*

*There are some feelings that you don’t want to admit (AK). We let some in, and then there were more (P6).*

Some said that they had made progress when it came with expressing their feelings:

*I’ve never talked about it. It may hurt and I may start weeping. Getting angry is something I never allow myself to do. But I’ve become much more conscious of it now (P5).*

Several expressed that when they started facing their feelings they became more honest and clear as a person, both to themselves and others:

*Shaking things up helps. I had an aha-experience (P5).*

*Instead of confronting him (husband) when I got mad at him, I would eat a bag of chips. Not only when I was at home, but wherever I was. However, telling others about it (the feeling), putting a name on it, has a special power in itself. A friend of mine became sour. It was tiresome and depressing. I had to tell her. She had not thought about it like that (P6).*

*I notice that I’ve changed. I didn’t like talking about how I looked before. That has changed. I’ve become more active (P3).*

What they spoke about

This theme revealed how the discussions in group therapy as well as the subsequent reflection put down in writing seemed to raise the participants’ awareness. Steering the discussion around a single feeling was a friendly but determined way to control the conversations. There was no room for much digression in the BEGP. Such control helped to identify what the individual thought about her/himself and become more aware of the meaning of feelings. Having to focus on feelings was demanding, but at the same time it was seen as something that strengthened the individual’s idea of her/himself.

### A More Generous Attitude Towards Oneself

The participants illuminated how the BEGP helped them to adopt a more generous attitude towards themselves. There was less black and white thinking connected to the fact that they had not succeeded in losing weight. Room for more nuances was developed:

*Seeing things in a longer perspective. Where have I come from and where am I now (P3)?*

*It was just a small setback. You’ve not fallen off. It’s not a disaster if you fail on one single day. Ok. It was one of those days. Tomorrow I’ll be back on track (P6).*

The BEGP also created recognition of oneself, one’s own process and ED:

*I’ve never said aloud that I have an overeating problem (P3).*

*It takes time to recognize that I actually do overeat. I feel better equipped to address my eating problem now. I notice that it helped a bit (P2).*

*An aha-experience. It felt good putting a name on things. Becoming more aware. It felt good stirring up things that had been suppressed for so long (P5).*

What they spoke about

Putting a name on their feelings helped. The participants had multiple experiences of not mastering a weight reduction program. Such failure led to a situation ruled by helplessness. The BEGP helped them find possible alternative thought patterns. They did not have to give up if they failed to succeed in meeting their expectations every single day, and setbacks were accepted as a part of life.

### On the Path to a Better Grip on the Eating Difficulties

Participation in the BEGP introduced changes in the way the participants handled their problematic eating. Writing helped them to gain control and structure, creating a change that led to improvement:

*I’ve learnt to throw away leftovers. Leftovers tend to create emotional havoc, that’s something I need to sort out. There was a period when I would go to the ice box when I got home from work and take out an ice cream. Then I thought, what are you doing? I ate the ice cream but then I started over again (P2).*

*I found my notebook and started writing. I’ve settled into the routine again. I take one small step at a time. I need structure. I’m ruled by the clock and write it down (P5).*

*I start thinking that I’ve eaten almost nothing today. But then I begin thinking it over. This is how much you’ve actually put into your mouth today (P1).*

*The days pass by in a mess. But I’m in control, and that helps (P6).*

The participants reflected over the impact writing had when it came to the meal diary, which was the homework given every week during the BEGP. The participants found the diary writing very confrontational and it had the potential to open up more for those who were dedicated to the task. Moreover, a more positive relationship to food and eating emerged:

*But I indulge myself on Saturdays (P6).*

*I enjoy eating food. Slowly but surely my weight is falling. It’ll be exciting to see (P2). It was good to know that this doesn’t mean I’m not allowed to eat this anymore (P3).*

The participants reflected on how hard it was to be honest about inner feelings, but the BEGP helped to initiate a process that became a way towards more openness in life. A new way of living was discerned:

*There are some feelings you don’t want to let in (P1).*

*But something happens. You let in a few and then there are more and more (P6). There has been a change. I have a plan when I go to the store (P3).*

*I don’t find it easy, but I feel quite happy inside and I’ve learnt a few things about how to handle it (P4).*

*I try to live in the present rather than floating along with one foot in the past and one in the future, not present in the here and now (P3).*

Some of them stated that they found writing hard as well. But afterwards it meant something to them:

*In the beginning, it was a struggle (P5).*

*I didn’t know what to write. I forced myself to write anything (P4).*

*It wasn’t easy. But it released quite a lot after I came home. It turned out very well (P6).*

What they spoke about

The participants reflected on how the step-by-step transformation took place. There were indications suggesting that it was easier to address and thereby control binge eating after the BEGP. It was as if they had gained a more positive grip on their BED, which helped them to lead a more satisfying life. The writing process had several impacts: in order to write one had to reflect and think about what to write; the written words were thereafter left behind to be reflected on in new ways for those who were willing to do so. It felt good to glimpse a way of coping, instead of resisting the process. The writing process was valuable, as it made them aware of what was happening, while at the same time helped to verify what happened yesterday and the day before, as well as setting the course ahead.

## Discussion

The aim of this pilot study was to evaluate patients’ experiences of attending a BEGP where therapeutic writing, affect consciousness, and cognitive therapy were integrated. The research question; how do patients evaluate their experience of attending an integrative binge eating group program? is answered by the three themes: enhanced self-awareness about the meaning of feelings, a more generous attitude towards oneself, and on the path to a better grip on the BED. The BEGP was evaluated and acknowledged as a response to the initial phases of therapeutic change and an essential part of the healing process. We will discuss the three themes in light of transition processes related to the BEGP. Emotional processing that includes the development of interventions enabling the establishment of a meaningful life appears to be important when facilitating transition processes (Meleis, 2010).The three themes were developed and interpreted in accordance with the participants’ own experiences. The approach, which is specially designed to fit a clinical environment, confirms the ideations of change over time, extends the repertoire of the BEGP, and increases our understanding of affect and therapeutic writing outcomes.

The first theme, *enhanced self-awareness of the meaning of feelings*, illuminates how writing as a therapeutic tool reconstructs and revises previous events that associate feelings with binge eating. This provides an opportunity to link a deeper meaning to broken threads of emotional situations in one’s life in the here and now. The focus on feelings in our program influences the awareness of one’s own body and food intake. An awareness of the meaning of these feelings is a start to understanding how to escape from negative and self-perpetuating binge eating cycles. Previous studies have related emotion regulation and emotional eating to a number of different states, such as boredom (Ferrell et al., 2020), food insecurity, perceived stress (Lopez-Cepero et al., 2019), food addiction, and irrational beliefs (Nolan & Jenkins, 2019). Dingemans et al. (2017) argued for the relevance of anger and sadness, along with negative relational and interpersonal emotional experiences such as being disappointed, hurt or feeling lonely. They further argued that the tendency to suppress such feelings, which is common in those with BED, leads to “increased psychopathology, thoughts and symptoms” (Dingemans et al., 2017, p. 1). The writing task presented in this pilot study is a way of making space for feelings and emotional awareness. Emotional expression facilitates cognitive processing of the problematic memory, which leads to effective psychological change (Smyth, 1998). As a result of being aware of the meaning of one’s own feelings, new sides of the self are revealed. These processes are supported by recent studies on third-wave therapies, which argue for targeting emotion regulation strategies in eating disorders (Linardon et al., 2017). Interventions where concealed and undisclosed feelings are verbalized in therapeutic processes are important and have been highlighted in previous studies on ED (Rørtveit & Severinsson, 2012). According to Lowe, therapeutic writing may be a helpful tool (Lowe, 2006) as it emerged from the psychotherapeutic tradition, where it is employed to relieve ailments associated with traumatic experiences (Smyth & Greenberg, 2000).

The second theme, *a more generous attitude towards oneself*, illuminates together with the first theme, how the start of a transition in BED must begin with awareness and self-generosity, which reflect one prerequisite of the transition process (Meleis, 2010). Developing a more generous attitude towards oneself as described in the present pilot study, is essential when dealing with undisclosed problematic feelings such as guilt, shame, or disgust. This is supported by Solbakken et al. (Solbakken et al., 2011), who claim that “unintegrated affect associated with mental representations of self and others may then cause a variety of problems, e.g., unclear self-experience or sense of self…” (p. 492). Self-awareness and understanding of mental states including feelings, cognitions, and intentions is a cognitive capacity emphasized as important for human psychological health and adaptive interpersonal functioning (Falkenström et al., 2014). One essential challenge illuminated in the present pilot study is to understand and support the ongoing transition processes. According to Meleis (2010), healthy transitions require a person to incorporate new knowledge to alter behavior and change the definition of self. The process leading to a more generous attitude to oneself as described by Solbakken et al. (2011) is necessary. To fully understand a transition process in binge eating, it is also essential to uncover and describe the effects of the true words. Words may be hidden behind binge eating processes (Rørtveit, 2014). Transitions are complex and multidimensional, hence may be easy to identify, or occur so slowly that they remain difficult to document (Meleis, 2010). Transitions related to binge eating problems are hard to document as changes under the surface are difficult to grasp. The theme a more generous attitude towards oneself takes into account that therapeutic processes are hard and demand deep reflections when trying to understand one’s own binge eating experiences. In this process, therapeutic writing may allow participants to more readily identify their cognitive distortions and replace them with more useful, rational thoughts. When such reflections are discussed despite the strong ambivalence towards change, a generous attitude towards oneself may be the next step.

The third theme, *on the path to a better grip on the eating difficulties*, is associated with lifestyle changes, despite the fact that the current BEGP is not. When a first step to understanding one’s eating patterns is achieved, a feeling of control and a better grip on binge eating emerge. CBT explains the cycle of dietary restraint and binge eating as the individuals’ attempt to control weight (Godfrey et al., 2015). Therefore, a better grip on the ED may provide a sense of increased control when establishing better eating patterns.

Our findings are in accordance with Solbakken et al. (Solbakken et al., 2011), who suggest that affect consciousness and integration of feelings in treatment are a predictor of change in psychotherapy. As the field of practice calls for research on psychological approaches to binge eating problems, interventions like the present one are valuable. Identifying process indicators that move clients in positive directions is important (Rørtveit, 2014). The therapeutic relationship is dependent on openness, dignity, and commitment to being reliable and honest. This is in line with our pilot study, as being on the path to a better grip on the ED is dependent on being really honest about painful experiences. Furnes and Dysvik (Furnes & Dysvik, 2011) state that expressive writing is a tool to discover an introspective technique and such writing has been shown to raise the writers’ awareness. Therapeutic writing employs processes of personal, explorative, and expressive awareness (Furnes & Dysvik, 2011). As such, and considering its power, therapeutic writing is suggested to be a support for people who experience various psychological difficulties (Baikie & Wilhelm, 2005; Furnes, 2008; B. Furnes & Dysvik, 2011, 2012).

One aspect of the BEGP is the type of therapy used and the themes detected, where the combination of affect consciousness and CBT provides an explicit focus on feelings at a deep level. Our pilot study indicates that situations in which contact with feelings is lost give rise to binge eating episodes. Furthermore, the pilot study describes how an explicit focus on the effects of therapy may lead to moments of realization. When combined with therapeutic writing, a space for mindful expression of thoughts and feelings is created.

## Conclusion

The BEGP described in this pilot study accords with concepts from cognitive therapy, affect consciousness, and therapeutic writing. This eclectic approach is adequate as a clinical intervention and our pilot study demonstrates that it is feasible in an outpatient psychiatric ward. We believe the three themes; enhanced self-awareness about the meaning of feelings, a more generous attitude towards oneself, and on the path to a better grip on the ED, are a necessary response to the initial phases of therapeutic change. We further conclude that the processes illuminated by these themes may be essential for healing by helping patients to find new words about their difficult and often silent binge eating condition.

### Implications

The results of this pilot study may provide knowledge for clinicians providing BEGP to prepare patients for the challenges of daily life. The results highlight important foci for patient education, education for peers and healthcare workers, for instance how to work with the psychological aspects of binge eating and feelings related to the condition. These results may also be valuable for those who work with patients suffering from obesity and those who seek help prior to or after bariatric surgery both in primary care and the hospital setting.

An implication for future studies is that important psychological variables should be identified and measured pre- and post-intervention in order to document specific effects. We suggest that the Emotional Eating Scale, comprises three subscales (namely anger, sadness, and anxiety) for measuring emotional eating (Arnow et al., 1995) be used as a quantitative measure in subsequent studies.

## Methodological Considerations

The method includes development and implementation in a naturalistic setting. It evaluates various ways in which the BEGP promotes reflection on the part of patients (Eriksson et al., 2007; Polit & Beck, 2004). Such a method highlights the process of user involvement, as the open-ended questions invite the patients to decide what to talk about. Qualitative evaluation will only give answers to questions about experiences and may not elicit hard facts. This aspect and the small number of participants in the present pilot study are limitations, thus the pilot study cannot be considered representative. Small scale pilot studies are carried out in order to improve larger projects, for instance the in terms of initial conceptualization (Polit & Hungler, 1995). Our pilot study bridges the gap between research and practice, which is in accordance with a qualitatively evaluated program (Morse et al., 2000). It provides more information about how to conceptualize affect-integration in a program over time, as experienced by the participants, thereby extending the repertoire of different intervention strategies (Morse et al., 2000). The pilot study could therefore be a precursor to future research and be strengthened by using several focus groups and individual interviews with participants who took part in the same program.
